# Identification of critical quality factors and critical satisfaction gaps in emergency training courses for new nurses by the multi-criteria network structure model

**DOI:** 10.1186/s12909-023-04330-0

**Published:** 2023-05-17

**Authors:** Wei-Ling Hu, Yen-Ching Chuang, Zhu Liduzi Jiesisibieke, Tao-Hsin Tung

**Affiliations:** 1 School of Medicine, Taizhou University, Taizhou, 318000 Zhejiang China; 2 Tarim Vocational and Technical College, Alar, Xinjiang 843300 China; 3 Business College, Taizhou University, Taizhou, 318000 Zhejiang China; 4 Institute of Public Health & Emergency Management, Taizhou University, Taizhou, 318000 Zhejiang China; 5 Key Laboratory of Evidence-Based Radiology of Taizhou, Linhai, 317000 Zhejiang China; 6 School of Public Health, Li Ka Shing Faculty of Medicine, The University of Hong Kong, Pokfulam, Hong Kong Special Administrative Region, China; 7 Evidence-Based Medicine Center, Taizhou Hospital of Zhejiang Province Affiliated to Wenzhou Medical University, Linhai, 317000 Zhejiang, China

**Keywords:** Emergency, New nurse, Training course, SERVQUAL, Decision-making trial and evaluation laboratory (DEMATEL), Importance-performance analysis (IPA), Multi-criteria decision-making (MCDM)

## Abstract

**Objective:**

To identify critical quality factors and critical satisfaction gaps in emergency training courses for new nurses through a systematic decision-making model.

**Methods:**

Firstly, the service quality (SERVQUAL) was used in the evaluation index system of this study. Then, the decision-making trial and evaluation laboratory (DEMATEL) method was used to analyze the relationship structure and the corresponding weights between the indicators. Finally, the importance-performance analysis (IPA) method was used to identify the categories of all indicators and the corresponding strategic directions. Fifteen new nurses in Taizhou Hospital of Zhejiang Province were selected as participants in this study.

**Results:**

The IPA results showed that “(*C*_13_),” “(*C*_22_),” “(*C*_52_),” “(*C*_53_),” “(*C*_54_),” “(*C*_55_),” “(*C*_56_),”and “(*C*_57_)” are critical satisfaction gaps. From the results of influence network and weight, empathy (*C*_5_) was the critical quality factor of the entire training course. The influence network relationship structure and weight had a 98.1% significant confidence level, indicating good stability.

**Conclusion:**

Teachers’ empathy is key to the learning outcomes of new nurses in emergency nursing training courses. Hence, teachers should be attentive to the empathetic quality of their teaching methods to help new nurses gain knowledge and experience in emergency care, especially when they come from different professions and departments.

## Introduction

Previous studies have paid little attention to the knowledge and skill readiness of new nurses in emergency nursing care environments [1]. For new nurses to be effective in the emergency department, it is necessary for them to receive professional nursing training on emergency knowledge and corresponding skills. Many studies have explored training course evaluations, such as emergency medicine undergraduate simulation training [2], advanced diploma courses [3], and psychiatric nursing [4]. However, few studies have evaluated the satisfaction of new nurses with the quality of emergency training courses.

Evaluating the satisfaction and quality improvement in training courses involves multiple criteria. Incorporating the practical experience of experts and managers can improve the accuracy of standard assessments and help identify the most influential criteria in their respective fields [5–8]. Multi-criteria decision-making (MCDM) is a modern scientific method used to evaluate, select, and improve alternatives based on an evaluation system that includes a set of qualitative and quantitative factors [5, 9–11]. Additionally, MCDM helps experts and managers balance and weigh factors to simplify their decision-making process [12, 13]. MCDM has also been widely used in decision-making problems in the medical and nursing fields, such as hospital performance [14], clinical decision-making [15], shared decision-making [16, 17], and nurses’ job satisfaction [9, 18]. The MCDM method is advantageous because it is based on the knowledge of experts. It also systematically provides evaluation and improvement to compensate for the limitations of decision-making in the evaluation and improvement of emergency training courses.

This study aimed to construct a hybrid multi-criteria decision-making model to assist hospital decision-makers and managers in evaluating the satisfaction with emergency training courses and systematically improving training quality during public health emergencies.

## Materials and methods

### Research design and modeling description

**Fig. 1:**
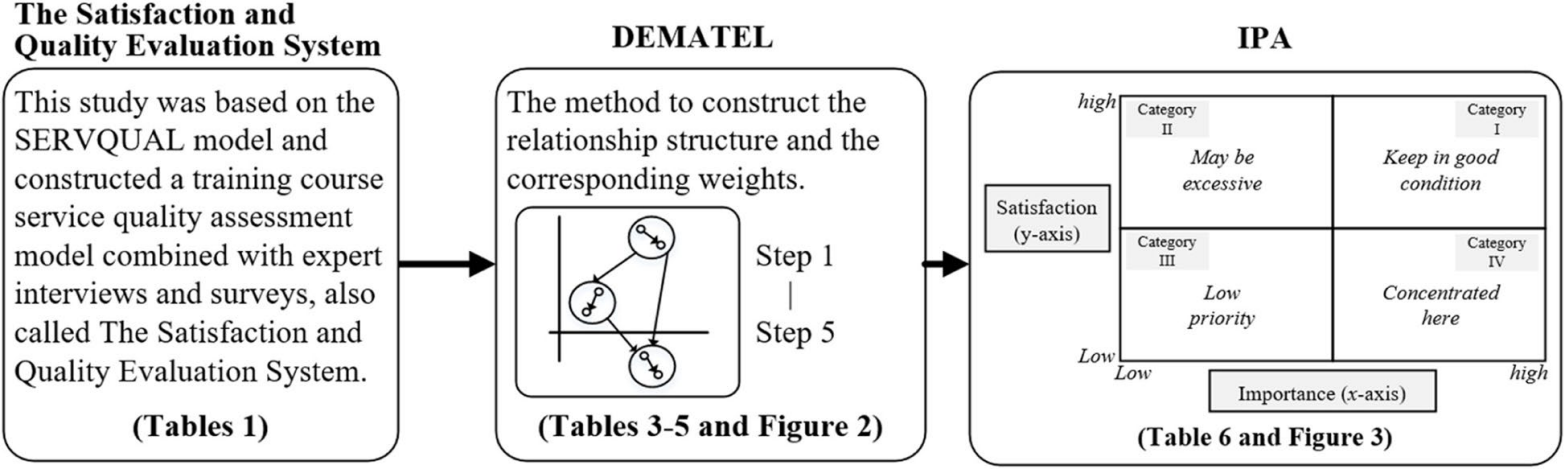
Decision modeling process

The decision model used in this study was based on three components. First, the evaluation model was designed based on the service quality (SERVQUAL) model. Then, we used the decision-making trial and evaluation laboratory (DEMATEL) method to construct the relationship structure between the criteria and their corresponding weights and further identify the critical quality factor. Finally, the importance-performance analysis (IPA) method was used to analyze the critical satisfaction gaps in emergency training courses. The research flow is illustrated in Fig. 1.

### The satisfaction and quality evaluation system

Parasuraman et al. formally proposed the SERVQUAL model theory in 1988 based on the research and development of the concept of service quality for measuring consumers’ perception of services [19]. The development process of this evaluation model was based on several theoretical models related to service quality and applies statistical analysis methods to analyze four survey objects/samples. The final version of the model consisted of five dimensions and 22 corresponding criteria, and its overall reliability was close to 0.9. Subsequently, the model was modified over time and applied to several service industries, such as retail, healthcare, e-commerce, tourism services, and other service sectors [20]. Additionally, a research review found that the model played a key role in research on medical service quality [21].

This study was based on the SERVQUAL model and constructed a quality assessment model for training course service combined with expert interviews and surveys, also known as The Satisfaction and Quality Evaluation System. Cronbach’s alpha values were 0.944 (goal), 0.856 (Tangibles *C*_1_), 0.881 (Reliability *C*_2_), 0.731 (Responsiveness *C*_3_), 0.753 (Assurance *C*_4_), and 0.923 (Empathy *C*_5_). Both the original scale and the results of this study show that the evaluation model has good reliability and can be used as the evaluation model in this study. In our model, the corresponding descriptions of the five dimensions are stated below, and the corresponding criteria are shown in Table 1.

- Tangibles (*C*_1_): Training courses provide corresponding tangible teaching facilities and equipment. This dimension includes four criteria.
- Reliability (*C*_2_): Provides a reliable teaching plan and corresponding training content to implement the promised training service accurately. This dimension includes five criteria.
- Responsiveness (*C*_3_): Teachers can provide timely training services and corresponding answers to questions. This dimension includes four criteria.
- Assurance (*C*_4_): Teachers’ knowledge and attitudes can earn students’ trust and confidence. This dimension includes four criteria.
- Empathy (*C*_5_): Teachers can provide care and personalized needs for students. This dimension includes seven criteria.

**Table 1:** Satisfaction and quality evaluation system of emergency training courses

Dimensions	Criteria
*C*_1_ Tangibility ($\alpha$ = 0.856)	*C*_11_ Modern teaching facilities
	*C*_12_ Teaching environment is attractive
	*C*_13_ Equipped with a professional teaching team
	*C*_14_ Teaching and learning facilities can support teaching and learning activities
*C*_2_ Reliability ($\alpha$ = 0.881)	*C*_21_ Teachers are able to complete lesson plans on time
	*C*_22_ Teachers are willing to help students with their learning difficulties
	*C*_23_ Teaching content is scientifically sound
	*C*_24_ Teachers are able to provide accurate instructional content
	*C*_25_ Teachers are able to appropriately evaluate learners’ performance (with a reasonable teaching evaluation system)
*C*_3_ Responsiveness ($\alpha$ = 0.731)	*C*_31_ Teachers are able to start and finish lessons on time
	*C*_32_ Teachers are able to answer learners’ questions in a timely manner
	*C*_33_ Teachers are able to help learners accomplish learning goals
	*C*_34_ Teachers are able to arrange the teaching schedule reasonably
*C*_4_ Assurance ($\alpha$ = 0.753)	*C*_41_ Teachers are trustworthy for their teaching capabilities
	*C*_42_ Teachers’ teaching process makes learners feel assured
	*C*_43_ Teachers are polite
	*C*_44_ Teachers are able to provide complete instructional services
*C*_5_ Empathy ($\alpha$ = 0.923)	*C*_51_ Teachers provide the same teaching services to every student
	*C*_52_ Teachers care for each student in the same manner
	*C*_53_ Teachers understand the learning needs of learners
	*C*_54_ Teachers take the benefits of learning for learners into account
	*C*_55_ Teachers are able to provide the class time that is acceptable to the students
	*C*_56_ Teachers care about learners’ enthusiasm for learning (self-confidence)
	*C*_57_ Teachers pay attention to learners’ progress

The overall internal consistency is 0.944 ($\alpha$ = 0.944)

### The DEMATEL technique

DEMATEL is an analytical technique developed by the Geneva Research Centre in 1973 to analyze the structure of influence relations among complex factors [22]. DEMATEL uses a structural modeling approach and represents the causal criteria [23], thus enabling decision-makers to obtain the causal relationships between criteria and use them as the main tool for problem-solving [24, 25]. The DEMATEL calculations are summarized as follows [26, 27]:

#### Step 1: Creating an average direct impact relationship matrix

Here, $k$^th^ represented each participant. The $k$^th^ respondent assessed the extent to which criterion i affected criterion j through a set of 5-point Likert scales (0 = no impact to 4 = very high impact). Next, an $n \times n$ matrix ${\varvec{A}}$ of non-negative values was obtained from the overall average, as shown in Eq. (1). Finally, the significant confidence level of the matrix ${\varvec{A}}$ can be confirmed by Eq. (2), with higher values representing higher stability/confidence results, where the threshold must exceed 95% (i.e., the average gap ratio is less than 5%).

1 $${\varvec{A}} = [a_{ij} ]_{n \times n} = \left[ {\frac{1}{Q}\sum\limits_{q = 1}^{Q} {d_{ij}^{q} } } \right]_{n \times n} , \, i,j \in \{ 1,2,...,n\}$$

2 $$\frac{1}{n(n - 1)}\sum\limits_{i = 1}^{k} {\sum\limits_{j = 1}^{k} {\frac{{\left| {a_{ij}^{k} - a_{ij}^{k - 1} } \right|}}{{a_{ij}^{k} }} \times 100\% } }$$

#### Step 2: Creating the normalized initial direct relation matrix

The initial direct-relation matrix ${\varvec{A}}$ can be normalized using Eq. (3) and (4) to obtain the normalized initial direct-relation matrix, ${\varvec{U}}$.

3 $$\rho = \mathop {\max }\limits_{i,j} \left[ {\max\nolimits_{i} \sum\nolimits_{j = 1}^{n} {a_{ij} } ,\max\nolimits_{j} \sum\nolimits_{i = 1}^{n} {a_{ij} } } \right]$$

4 $${\varvec{U}} = \frac{{\varvec{A}}}{\rho }$$

Here, the values of the matrix ${\varvec{U}}$ are all ratios between 0 and 1.

#### Step 3: Creating the total relation matrix

The total relation matrix ${\varvec{T}}$ is obtained using the normalized initial direct-relation matrix ${\varvec{U}}$ using Eq. (5).

5 $${\varvec{T}} = {\varvec{U}} + {\varvec{U}}^{2} + \cdots + {\varvec{U}}^{\Delta } = {\varvec{U}}({\varvec{I}} - {\varvec{U}})^{ - 1} ,{\text{when}}\mathop {\lim }\limits_{\Delta \to \infty } {\varvec{U}}^{\Delta } = [0]_{n \times n}$$

Here, the matrix ${\varvec{I}}$ is a unit matrix with diagonal of 1, and the rest are 0.

#### Step 4: Obtain the influential network relation map

The “exerted influence ($r_{i}$)” and “received influence ($s_{i}$)” of each criterion were obtained through Eqs. (6) and (7), respectively:

6 $$r_{i} = \left( {r_{i} } \right)_{{_{n \times 1} }} = (r_{1} ,...,r_{i} ,...,r_{n} ) = \left[ {\sum\nolimits_{j = 1}^{n} {t_{ij} } } \right]_{n \times 1}^{{}}$$

7 $$s_{i} = \left( {s_{i} } \right)_{{_{n \times 1} }} = \left( {s_{j} } \right)_{1 \times n}^{^{\prime}} = (s_{1} ,...,s_{j} ,...,s_{n} )^{^{\prime}} = \left[ {\sum\nolimits_{i = 1}^{n} {t_{ij} } } \right]_{1 \times n}^{^{\prime}}$$

The “significance ($r_{i} + s_{i}$)” of each criterion represented the degree of significance of the relative influence of that criterion in relation to other criteria, where higher values represented a more significant influence caused to the system. In contrast, the “relationship ($r_{i} - s_{i}$)” represented the nature of the criterion’s significant influence within the system, where a positive value meant that the criterion is of the nature of “exerting influence” ($r_{i}$) on others. These criteria were grouped together and were called the positive group. On the other hand, a negative value meant that the criterion was of the nature of “receiving influence” ($s_{i}$), which was also called the negative group.

#### Step 5: Determining the influence relation weights for the criteria

The influence relation weight of each criterion was obtained through Eq. (8).

8 $$w_{i} = \frac{{\left( {r_{i} + s_{i} } \right)}}{{\sum\nolimits_{i = 1}^{n} {\left( {r_{i} + s_{i} } \right)} }}$$

### The IPA method

Martilla and James developed IPA in 1997 to identify key performance criteria for a product or service [28–30]. The method helps decision-makers easily understand the performance and importance of all criteria and further identify the critical ones. This method categorizes all criteria into the following four categories based on performance and importance scores [31, 32]. In this study, the weight results of the DEMEL method was used as the importance (i.e., *x*-axis). Then, during the questionnaire, the satisfaction obtained was used as performance (i.e., *y*-axis). The average of both these weights and satisfaction was used as the central point to assist in the classification of all criteria. The four categories and their corresponding decisions are described below.

- Category I: Highly relevant (high importance and high performance). These criteria are important for teaching content, services, and training courses. They are essential for training courses, and the performance evaluation after courses is high. Hospital administrators can be assured of these criteria for the time being.
- Category II: May be excessive (low importance and high performance). These criteria are less important for teaching content, services, or training courses. They are not important for training courses, and the performance evaluation after courses is high. Hospital administrators may ignore these criteria for the time being.
- Category III: Low priority (low importance and low performance). These criteria are the least important factors for teaching content, services, and training courses. They are not important for training courses, and their performance evaluation after courses is very low. Compared with other categories, hospital administrators may not need to address these criteria for the time being.
- Category IV: Administratively prioritized (high importance and low performance). These criteria are the most important improvement factors for teaching content, services, and training courses. These are important for training courses; however, their performance evaluations after the courses are very low. Compared with other categories, it is most important for hospital administrators to prioritize these criteria. This category was also called critical satisfaction gaps in this study.

### Ethical approval

All courses were conducted following the guidelines of our institution’s ethics committee and according to the principles of the Declaration of Helsinki. All participant data were anonymous. Institutional Review Board (IRB) of Taizhou University, Taizhou, China (ID: TZYXY2021-423) approved the oral informed consent procedure of this study and the whole study.

### Data collection and description

The recruitment was conducted after approval from the Institutional Review Board (IRB) of Taizhou University, and the head of the hospital and department. The participants were recruited through referrals from the department head. Before conducting the research, we explained all the procedures and criteria to participants, and stated that their information would be confidential. The investigation period of this study was from April 11, 2021, to April 20, 2021. In total, 15 new nurses (i.e., working service years less than 1 year) participated. All participants provided informed consent.

The content of the questionnaire was divided into three parts: first, the degree of interaction between the estimation criteria; second was to investigate the satisfaction level of the course; and third was the background of the interviewees. The data of this study were calculated by excel and SPSS; the former was to calculate the results (i.e., structure and weight) of the DEMTEL method, and the latter was to calculate the results of the IPA method. All participants were under 30 years of age. Among them, 87% were women, 60% had bachelor’s degrees, and their participating majors were distributed in the emergency, internal medicine, surgery, and nursing departments. The background and descriptions of the respondents in this study are shown in Table 2.

**Table 2:** Background and characteristics of respondents in this study

Characteristics	*n* (%)
Gender	
Male	2 (13%)
Female	13 (87%)
Education	
Specialty	5 (33%)
Bachelor	9 (60%)
Master or above	1 (7%)
Age	
≤ 30	15 (100%)
Department	
Emergency	4 (27%)
Internal medicine	5 (33%)
Surgery	3 (20%)
Nursing department	3 (20%)
Years of service	
≤ 1	15 (100%)

## Results

### Results of influence structure

Table 3 shows the structure of the influence relationship between criteria from the clinical experience of 15 participants and forms an average direct influence impact matrix. For this matrix, the mean ratio of the variance was 0.0451 (i.e., 4.52%, less than 5%), and the significance level was 95.48% (i.e., more than 95%). This result also shows that the experience gap of this group of respondents was within the acceptable range, and that the network structure results had good stability. In other words, the matrix represents a common empirical consensus among this group of experts. Subsequent additions of relevant experts showed little change in results.

**Table 3:** Average direct impact matrix of 15 respondents

	*C*_11_	*C*_12_	*C*_13_	*C*_14_	*C*_21_	*C*_22_	*C*_23_	*C*_24_	*C*_25_	*C*_31_	*C*_32_	*C*_33_	*C*_34_	*C*_41_	*C*_42_	*C*_43_	*C*_44_	*C*_51_	*C*_52_	*C*_53_	*C*_54_	*C*_55_	*C*_56_	*C*_57_
*C*_11_	0.00	2.80	2.27	3.20	1.80	1.53	2.13	2.00	1.60	1.33	1.73	1.80	1.67	1.40	1.40	1.20	1.93	1.27	1.13	1.47	1.33	1.33	1.40	1.47
*C*_12_	1.73	0.00	2.00	1.93	2.07	1.47	1.73	1.67	1.67	1.47	1.40	1.47	1.47	1.13	1.60	1.20	1.60	1.27	1.27	1.53	1.27	1.40	1.40	1.40
*C*_13_	1.73	1.60	0.00	2.20	3.00	2.33	2.93	2.80	2.33	2.00	2.73	2.93	2.80	2.80	2.87	1.80	2.87	2.20	1.93	2.67	2.80	2.27	2.20	2.53
*C*_14_	2.20	2.20	1.80	0.00	2.33	1.60	2.33	2.07	1.53	1.60	1.80	2.07	1.93	1.33	1.60	1.20	1.93	1.47	1.20	1.47	1.67	1.40	1.33	1.27
*C*_21_	1.13	0.87	2.00	1.47	0.00	1.87	2.07	2.33	2.47	2.73	2.20	2.07	2.47	2.00	2.20	2.00	2.33	1.80	1.73	1.87	2.20	2.47	1.53	2.00
*C*_22_	1.33	1.53	2.20	1.53	2.40	0.00	2.13	2.53	2.67	2.20	3.00	2.80	2.73	2.60	3.13	2.07	2.87	2.47	2.67	3.00	3.00	2.20	2.80	2.93
*C*_23_	1.40	1.40	2.33	1.33	2.07	1.67	0.00	2.33	2.00	2.13	2.00	2.53	2.67	2.40	2.40	1.80	2.33	2.00	2.07	2.67	2.47	2.47	1.93	2.40
*C*_24_	1.33	1.33	2.07	1.47	2.47	2.00	2.60	0.00	2.40	1.80	2.33	2.27	2.53	2.33	2.40	1.87	2.53	2.67	2.07	2.27	2.53	2.47	2.27	2.07
*C*_25_	0.93	1.33	2.13	1.27	1.73	2.07	2.00	2.07	0.00	1.80	2.00	2.20	2.33	2.33	2.33	2.00	2.33	2.33	2.53	2.60	2.60	2.27	2.47	2.47
*C*_31_	1.33	1.47	2.13	1.47	2.33	2.13	2.20	2.33	2.40	0.00	2.07	2.53	2.93	2.47	2.47	2.07	2.40	2.47	2.27	2.07	2.00	2.33	2.20	2.33
*C*_32_	1.40	1.67	2.33	2.00	2.80	2.87	2.47	2.60	2.80	2.53	0.00	2.67	2.93	2.93	3.20	2.67	2.93	2.80	2.80	3.07	2.93	2.73	2.93	3.00
*C*_33_	0.87	1.27	2.13	1.53	2.13	2.20	2.47	2.60	2.53	1.93	2.33	0.00	2.67	2.33	2.13	2.20	2.20	2.47	2.00	2.40	2.60	2.40	2.40	2.40
*C*_34_	0.87	1.40	1.80	1.20	2.60	2.27	2.13	2.53	2.13	2.47	2.40	2.53	0.00	2.20	2.27	2.00	2.20	2.20	2.27	2.40	2.33	2.53	2.40	2.13
*C*_41_	0.93	1.73	2.47	1.33	2.27	2.40	2.80	2.47	2.27	2.07	2.80	2.80	2.53	0.00	2.87	2.33	2.73	2.47	2.33	2.53	2.47	2.47	2.53	2.73
*C*_42_	1.40	2.07	2.40	1.60	2.47	2.73	2.67	2.27	2.87	2.40	2.80	2.73	2.80	3.27	0.00	2.80	3.00	2.73	2.67	2.93	3.00	2.60	2.93	3.00
*C*_43_	1.07	1.80	2.40	1.60	2.00	2.47	2.07	2.07	2.20	2.40	2.33	2.67	2.00	2.47	2.53	0.00	2.67	2.73	2.87	2.80	2.47	2.53	2.53	2.60
*C*_44_	1.27	1.73	2.47	1.33	2.53	2.27	2.47	2.33	2.67	1.87	2.40	2.40	2.53	2.60	2.47	2.13	0.00	2.47	2.27	2.40	2.40	2.47	2.40	2.60
*C*_51_	1.47	1.93	2.60	1.67	2.60	2.80	2.60	2.47	2.93	1.87	2.53	2.67	2.47	2.93	3.20	2.53	2.80	0.00	2.67	3.07	3.00	3.93	3.00	2.87
*C*_52_	1.27	1.93	2.60	1.60	2.20	2.73	2.33	2.07	2.80	2.20	2.67	2.67	2.40	2.67	2.80	2.60	2.93	3.13	0.00	2.87	2.80	2.47	2.80	2.60
*C*_53_	1.20	1.93	2.73	1.60	2.47	2.60	2.80	2.53	2.47	2.07	2.87	2.87	2.67	2.93	3.00	2.47	2.73	2.53	2.73	0.00	3.07	2.67	2.80	2.87
*C*_54_	1.27	1.87	2.60	1.40	2.40	2.73	2.47	2.47	2.67	2.47	2.80	3.00	2.40	2.87	2.87	2.40	2.67	2.80	2.73	2.67	0.00	2.67	2.67	2.87
*C*_55_	1.20	1.87	2.47	1.40	2.67	2.13	2.60	2.33	2.93	2.53	2.33	2.53	2.40	2.33	2.60	2.27	2.33	2.53	2.33	2.67	2.73	0.00	2.60	2.67
*C*_56_	1.33	2.20	2.67	1.80	2.47	2.87	2.60	2.67	2.93	2.60	2.80	3.00	2.47	3.07	3.13	2.67	2.67	2.73	3.00	3.07	2.93	2.80	0.00	3.00
*C*_57_	1.20	1.80	2.47	1.40	2.53	2.73	2.40	2.27	2.67	2.07	2.60	2.73	2.60	2.73	3.00	2.53	2.67	2.53	2.53	2.87	2.80	2.67	2.73	0.00

The average variance ratio of respondents’ data ($\frac{1}{n(n - 1)}\sum\limits_{i = 1}^{k} {\sum\limits_{j = 1}^{k} {\frac{{\left| {a_{ij}^{k} - a_{ij}^{k - 1} } \right|}}{{a_{ij}^{k} }} \times 100\% } }$) is 4.52% (less than 5%). In other words, the significant confidence level reached 95.48% (i.e. greater than 95%)

The average direct influence matrix (${\varvec{A}}$) further obtained four indicators for all dimensions/criteria through the calculation of a series of mathematical equations (i.e., Eqs. (3, 4, 5, 6, 7)). Table 4 shows “exerted influence” ($r_{i}$), “received influence” ($s_{i}$), “significance” ($r_{i} + s_{i}$), and “relation” ($r_{i} - s_{i}$) data for all dimensions and criteria. In the dimension level, the “significance” ($r_{i} + s_{i}$) from high to low was $C_5\;\mathrm f\;C_4\;\mathrm f\;C_3\;\mathrm f\;C_2\;\mathrm f\;C_1$. In addition, from the perspective of “relation” ($r_{i} - s_{i}$), the dimensions belonging to the positive group were “($C_{1}$)” and “($C_{5}$).” In contrast, “($C_{2}$),” “($C_{3}$),” and “($C_{4}$)” belonged to the negative group. In the criteria of “(*C*_1_),” “significance” ($r_{i} + s_{i}$) from high to low was $C_{13}\;\mathrm f\;C_{14}\;\mathrm f\;C_{12}\;\mathrm f\;C_{11}$. In addition, from the perspective of the “relation” ($r_{i} - s_{i}$), the criteria belonging to the positive group were “($C_{11}$)” and “($C_{13}$).” In contrast, “($C_{12}$)” and “($C_{14}$)” belonged to the negative group.

**Table 4:** The influence structure for dimensions and criteria

Dimensions /Criteria	“Exerted influence ($r_{i}$)”	“Received influence ($s_{i}$)”	Significance ($r_{i} + s_{i}$)	Relationship ($r_{i} - s_{i}$)	group
*C*_1_	1.033	0.968	2.001	0.065	+
* C*_11_	4.559	3.530	8.089	1.030	+
* C*_12_	4.158	4.668	8.826	-0.510	-
* C*_13_	6.675	6.293	12.968	0.382	+
* C*_14_	4.615	4.361	8.976	0.254	+
*C*_2_	1.234	1.329	2.562	-0.095	*-*
* C*_21_	5.488	6.443	11.931	-0.955	-
* C*_22_	6.805	6.281	13.086	0.525	+
* C*_23_	5.841	6.523	12.364	-0.682	-
* C*_24_	5.995	6.393	12.387	-0.398	-
* C*_25_	5.811	6.684	12.495	-0.874	-
*C*_3_	1.298	1.333	2.631	-0.036	*-*
* C*_31_	6.018	5.815	11.834	0.203	+
* C*_32_	7.272	6.560	13.831	0.712	+
* C*_33_	6.026	6.894	12.920	-0.867	-
* C*_34_	5.918	6.724	12.641	-0.806	-
*C*_4_	1.354	1.356	2.711	-0.002	*-*
* C*_41_	6.512	6.741	13.253	-0.228	-
* C*_42_	7.160	6.984	14.145	0.176	+
* C*_43_	6.390	5.887	12.277	0.503	+
* C*_44_	6.271	6.853	13.124	-0.582	-
*C*_5_	1.430	1.363	2.793	0.068	+
* C*_51_	7.224	6.487	13.711	0.738	+
* C*_52_	6.834	6.268	13.102	0.566	+
* C*_53_	6.995	6.858	13.854	0.137	+
* C*_54_	6.899	6.879	13.778	0.020	+
* C*_55_	6.498	6.660	13.158	-0.162	-
* C*_56_	7.310	6.514	13.824	0.796	+
* C*_57_	6.763	6.739	13.503	0.024	+

1. “Significance ($r_{i} + s_{i}$)” represented the degree of significance of the relative influence of that criterion in relation to other criteria

2. “Relationship ($r_{i} - s_{i}$)” represented the nature of the criterion’s significant influence within the system, where a higher positive value ( +) means In contrast, the criterion is of the nature of “exerting influence” ($r_{i}$) on others; on the other hand, a negative value (-) means that the criterion is of the nature of “receiving influence” ($s_{i}$)

3. ( +) is positive group; (-) is negative group

In the criteria of “($C_{2}$),” “significance” ($r_{i} + s_{i}$) from high to low was $C_{22}\;\mathrm f\;C_{25}\;\mathrm f\;C_{24}\;\mathrm f\;C_{23}\;\mathrm f\;C_{21}$. In addition, from the perspective of the “relation” ($r_{i} - s_{i}$), the criterion belonging to the positive group was “($C_{22}$).” The other criteria were assigned to the negative group. In the criteria of “($C_{3}$),” “significance” ($r_{i} + s_{i}$) from high to low was $C_{32\;}\mathrm f\;C_{33}\;\mathrm f\;C_{34}\;\mathrm f\;C_{31}$. In addition, from the perspective of the “relation ($r_{i} - s_{i}$),” the criteria belonging to the positive group were “(*C*_31_)” and “($C_{32}$).” In contrast, “($C_{33}$)” and “($C_{34}$)” belonged to the negative group.

In the criteria of “($C_{4}$),” “significance” ($r_{i} + s_{i}$) from high to low was $C_{42}\;\mathrm f\;C_{41}\;\mathrm f\;C_{44}\;\mathrm f\;C_{43}$. In addition, from the perspective of the “relation” ($r_{i} - s_{i}$), the criteria belonging to the positive group were “(*C*_42_)” and “($C_{43}$).” In contrast, “($C_{41}$)” and “($C_{44}$)” belonged to the negative group. In the criteria of “($C_{5}$),” “significance” ($r_{i} + s_{i}$) from high to low was $C_{53}\;\mathrm f\;C_{56}\;\mathrm f\;C_{54}\;\mathrm f\;C_{51}\;\mathrm f\;C_{57}\;\mathrm f\;C_{55}\;\mathrm f\;C_{52}$. In addition, from the perspective of the “relation” ($r_{i} - s_{i}$), the criterion belonging to the negative group was “($C_{55}$).” The other criteria were assigned to the positive group.

### Results of influence weights

The “significance” ($r_{i} + s_{i}$) of dimensions and criteria was transformed into a set of influence weights by Eq. (8). Table 5 shows the influence weights of all dimensions and the corresponding criteria, which represent the degree of influence weight of this factor in the entire evaluation system. The weight can be further divided into local point of view and overall point of view. Local weight and ranking referred to the relative importance of the dimensions/criteria in local views and the corresponding ranking. Global weight and ranking referred to the relative importance of the criterion in all criteria (regardless of local views) and the corresponding ranking.

**Table 5:** The influence weights for dimensions and criteria

Dimensions/Criteria	Local weight	Local ranking	Global weight	Global ranking
*C*_1_	0.158	5		
* C*_11_	0.171	4	0.027	24
* C*_12_	0.187	3	0.029	23
* C*_13_	0.274	1	0.043	13
* C*_14_	0.190	2	0.030	22
*C*_2_	0.202	4		
* C*_21_	0.197	5	0.040	20
* C*_22_	0.216	1	0.044	12
* C*_23_	0.204	4	0.041	18
* C*_24_	0.205	3	0.041	17
* C*_25_	0.206	2	0.042	16
*C*_3_	0.207	3		
* C*_31_	0.190	4	0.039	21
* C*_32_	0.222	1	0.046	3
* C*_33_	0.208	2	0.043	14
* C*_34_	0.203	3	0.042	15
*C*_4_	0.213	2		
* C*_41_	0.207	2	0.044	8
* C*_42_	0.221	1	0.047	1
* C*_43_	0.192	4	0.041	19
* C*_44_	0.205	3	0.044	10
*C*_5_	0.220	1		
* C*_51_	0.208	4	0.046	6
* C*_52_	0.199	7	0.044	11
* C*_53_	0.210	1	0.046	2
* C*_54_	0.209	3	0.046	5
* C*_55_	0.199	6	0.044	9
* C*_56_	0.209	2	0.046	4
* C*_57_	0.205	5	0.045	7

1. Local weight and ranking refer to the relative importance of dimensions/criteria in local views and the corresponding ranking

2. Global weight and ranking refer to the relative importance of the criterion in all criteria (regardless of Local views) and the corresponding ranking

The higher the value, the more attention should be paid to the dimension or criterion in subsequent IPA analysis. The dimensions “($C_{5}$),” “($C_{4}$),” and “($C_{3}$)” were the top three with the highest weight compared with other dimensions. In each dimension, “($C_{13}$),” “($C_{22}$),” “($C_{32}$),” “($C_{42}$),” and “($C_{53}$)” were the criteria with the highest weight. In addition, from the overall perspective, “($C_{42}$),” “($C_{53}$)” and “($C_{32}$),” were the top three criteria.

### Results of IPA evaluation

Table 6 shows the importance-satisfaction analysis of the emergency training courses for the 15 respondents. All criteria can be roughly classified into four categories using the IPA. The criteria in Category I (Highly relevant) were “($C_{32}$)”, “($C_{33}$)”, “($C_{34}$)”, “($C_{41}$)”, “($C_{42}$)”, “($C_{44}$)”, and “($C_{51}$)”. The criteria in Category II (May be excessive) were “($C_{21}$)”, “($C_{24}$)”, “($C_{31}$)”, and “($C_{43}$)”. The criteria in Category III (Low priority) were “($C_{11}$)”, “($C_{12}$)”, “($C_{14}$)”, “($C_{23}$)”, and “($C_{25}$)”. The criteria in Category IV (Administratively prioritized) were “($C_{13}$)”, “($C_{22}$)”, “($C_{52}$)”, “($C_{53}$)”, “($C_{54}$)”, “($C_{55}$)”, “($C_{56}$)”, and “($C_{57}$)”.

**Table 6:** The importance-satisfaction analysis of the emergency training courses

Criteria	Global weight	Performance	Group
*C*_11_ Modern teaching facilities	0.027	3.800	III
*C*_12_ Teaching environment is attractive	0.029	3.867	III
*C*_13_ Equipped with a professional teaching team	0.043	4.133	IV
*C*_14_ Teaching and learning facilities can support teaching and learning activities	0.030	4.067	III
*C*_21_ Teachers are able to complete lesson plans on time	0.040	4.400	II
*C*_22_ Teachers are willing to help students with their difficulties of learning	0.044	4.133	IV
*C*_23_ Teaching content is scientifically sound	0.041	4.133	III
*C*_24_ Teachers are able to provide accurate instructional content	0.041	4.267	II
*C*_25_ Teachers are able to appropriately evaluate learners’ performance (with a reasonable teaching evaluation system)	0.042	3.933	III
*C*_31_ Teachers are able to start and finish lessons on time	0.039	4.533	II
*C*_32_ Teachers are able to answer learners’ questions in a timely manner	0.046	4.600	I
*C*_33_ Teachers are able to help learners accomplish learning goals	0.043	4.267	I
*C*_34_ Teachers are able to reasonably arrange the teaching schedule	0.042	4.400	I
*C*_41_ Teachers are trustworthy for their teaching capabilities	0.044	4.333	I
*C*_42_ Teachers’ teaching process makes learners feel assured	0.047	4.200	I
*C*_43_ Teachers are polite	0.041	4.733	II
*C*_44_ Teachers are able to provide complete instructional services	0.044	4.267	I
*C*_51_ Teachers provide the same teaching services to every student	0.046	4.200	I
*C*_52_ Teachers care for each student in the same manner	0.044	4.000	IV
*C*_53_ Teachers understand the learning needs of learners	0.046	3.800	IV
*C*_54_ Teachers takes the benefits of learning for learners into account	0.046	3.933	IV
*C*_55_ Teachers are able to provide the class time that is acceptable to the students	0.044	4.133	IV
*C*_56_ Teachers care about learners’ enthusiasm for learning (self-confidence)	0.046	3.867	IV
*C*_57_ Teachers pay attention to learners’ progress	0.045	4.000	IV

## Discussions

### Influential structure analysis

**Fig. 2:**
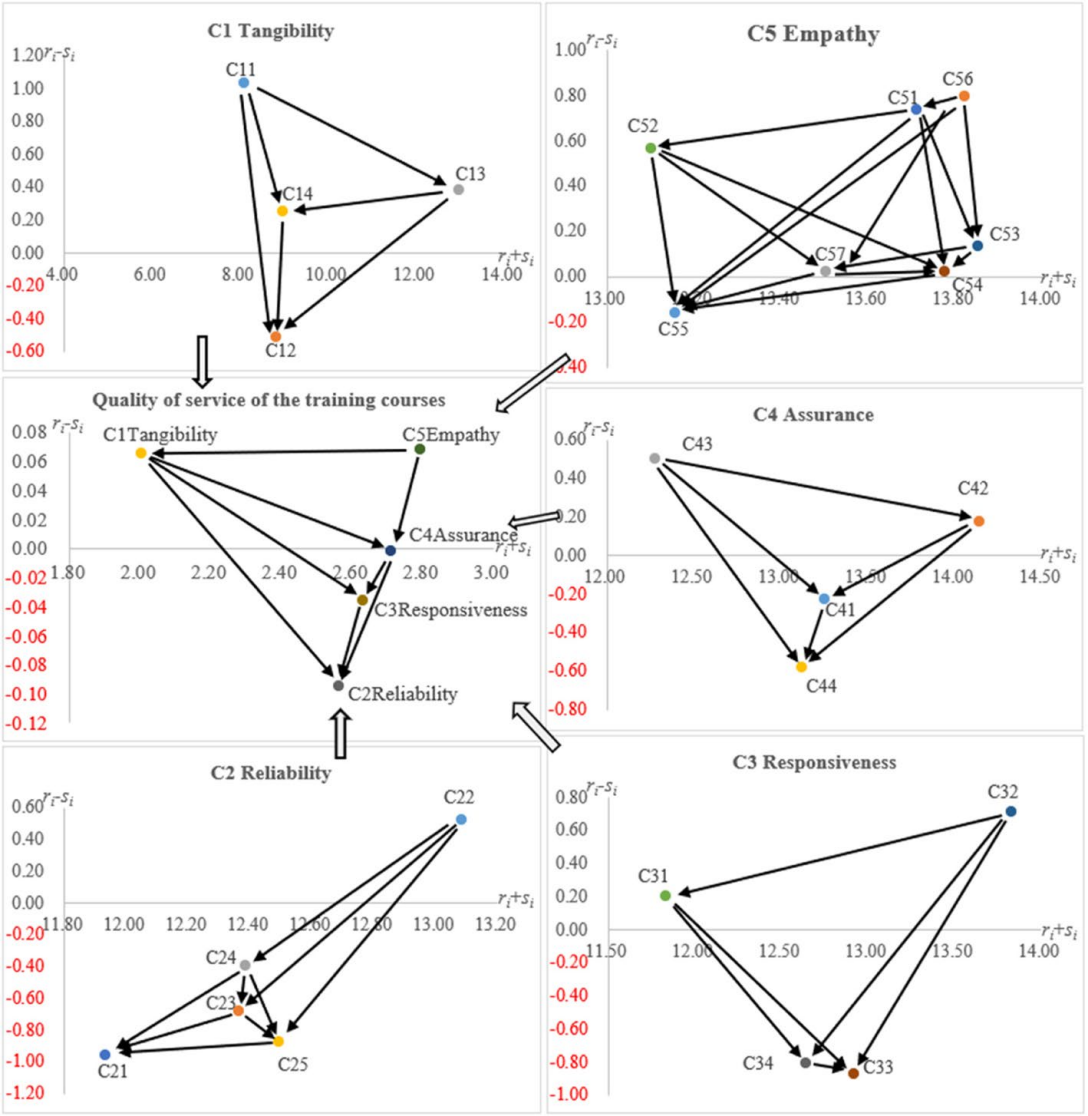
Influential network-relation map

Figure 2 shows the structure of the influence relations between all dimensions and criteria levels.

In the dimensions, “($C_{5}$)” was the main source of influence on the other dimensions. An empathic teacher would consider the students’ perspectives to understand the work situation and learning constraints of new nurses from different departments and thus design an appropriate training curriculum and teaching plan [19]. Additionally, the teacher should be able to use the teaching facilities and environment during the teaching process. In turn, the teacher would maintain a good professional image and inspire new nurses’ interest and confidence in learning. Ultimately, new nurses who participated in the revised training course may find the content and process of this training course reliable.

In the criteria of “($C_{1}$),” “($C_{11}$)” was the main influence criterion. Good physical facilities are essential elements of a teaching and learning environment. Usually, hospitals invite renowned external scholars or experts to conduct training, and the training venue and teaching resources are located in the hospital. Moreover, most of the training are scheduled outside working hours, such as weekday evenings, Saturdays, and Sundays. Therefore, good teaching facilities in the hospital facilitated teachers’ provision of professional emergency knowledge and training. It created an attractive teaching environment and motivated new nurses to attend training courses during their time off from work and enjoy the atmosphere of the learning environment.

The results of a past study [33] also point out that proper facility management creates a favorable learning environment. For the instructor, good care management helps coordinate classroom activities; for the students, it effectively instills maintenance skills.

In the criteria of “($C_{2}$),” “($C_{22}$)” was the main influence criterion. All the nurses in the study were at the hospital for 1 year or less. When a teacher is willing to help new nurses with difficulties during the training process, they pay attention to the learning status of each new nurse to provide appropriate teaching content and properly assess the student’s learning performance. The teacher should also ensure that the content is scientifically sound and effective and that the lesson plan is completed on time.

In the criteria of “($C_{3}$),” “($C_{32}$)” was the main influence criterion. New nurses who started learning emergency nursing faced difficulties and problems in learning at the beginning. If teachers could answer new nurses’ questions promptly, start and end classes on time, and appropriately arrange the teaching schedule, it may be easier to help students achieve their learning goals with a smooth training process.

In the criteria of “($C_{4}$),” “($C_{43}$)” was the main influence criterion. A polite teacher respects the learning status of each new nurse and communicates and discusses the content with them. During the teaching process, new nurses can express themselves confidently and feel assured of the teacher’s teaching capabilities. This teaching atmosphere helps teachers to deliver complete content and services successfully.

In the criteria of “($C_{5}$),” “($C_{56}$)” was the main influence criterion. Enthusiasm for learning is an essential element for completing the learning process. Teachers should be concerned about each new nurse’s learning status and provide the same teaching services and care. Teachers should also understand the learning needs, progress, and outcomes of new nurses. Finally, teachers should be able to schedule classes that are acceptable to new nurses.

### Influential weight analysis

Table 5 shows that the dimension with the highest weight was “($C_{5}$).” A teacher with empathy can better consider trainees’ learning needs from their perspectives, observe the benefits of their learning, and dynamically monitor their learning progress. When new nurses encounter conflicts between training and work, teachers should be able to adjust their schedules to offer a class time that is acceptable to trainees on time. During the process of nursing training, new nurses may encounter many learning obstacles that require teachers to pay special attention to their learning status and assist them in solving their learning problems in a timely manner. Ultimately, new nurses would be more willing to learn and complete the training course in emergency nursing. Moreover, this study had the same results as those of a previous study, [34] in which empathy had a positive impact on students’ professional development and learning environment in nursing. Because of this, faculty should be strongly encouraged to place more emphasis on the principles of empathy and care in nursing, which would involve good communication with students.

From the perspective of criteria in each dimension, the criterion with the highest weight in “($C_{1}$)” was “($C_{13}$).” A teaching team with extensive expertise and experience can better use modern teaching tools, provide a better quality of teaching, and make the content better accepted by new nurses to ensure the quality of teaching altogether.

In “($C_{2}$),” “($C_{22}$)” was the highest weighted criterion. New nurses come from different departments with different learning capabilities and might encounter difficulties during the learning process. Teachers should accurately understand students’ specific difficulties and then, appropriately arrange the teaching content and design a reasonable evaluation system that is achievable through the trainees’ efforts to ensure the quality of learning of new nurses.

In “($C_{3}$),” “($C_{32}$)” was the criterion with the highest weight. When learners encounter learning problems, the teacher should be able to answer them promptly, allowing the teaching progress to proceed as scheduled. Since students can grasp the key points in class and review them thoroughly after class, the teaching and learning activities proceed smoothly.

In the “($C_{4}$),” “($C_{42}$)” was the highest weighted criterion. New nurses were still getting familiar with the hospital, were unfamiliar with the teacher, and had even less knowledge about emergency care. Consequently, new nurses often fear that they will not be able to meet the learning objectives of the training course, causing worry. Therefore, teachers should inform new nurses about the learning objectives and trajectory and provide learning methods in advance. Meanwhile, daily communication with trainees should be increased to gain trust and reassure them about the training process of this emergency nursing course.

Finally, in “($C_{5}$),” “($C_{53}$)” was the highest-weighted criterion. New nurses from different departments might have had different clinical nursing experiences and learning needs. The teacher should understand the learning needs of each trainee on time and schedule the lecture time appropriately based on the work schedule of new nurses so that each trainee can attend the lecture. During the training process, teachers should monitor new nurses’ learning progress and learning outcomes and promptly encourage students who lack confidence. All of the above are necessary measures and behaviors.

### Systematic improvement strategy from the influential network perspective

Table 6 shows that Category IV included “($C_{13}$)”, “($C_{22}$)”, “($C_{52}$)”, “($C_{53}$)”, “($C_{54}$)”, “($C_{55}$)”, “($C_{56}$)”, and “($C_{57}$)”. Thus, the main problem with this training course was that teachers lack empathy for new nurses. In the training process, teachers failed to consider the learning needs, learning conditions, and learning difficulties from their point of view. The primary reason is that they come from different nursing majors, and everyone had different abilities regarding emergency nursing content.

**Fig. 3:**
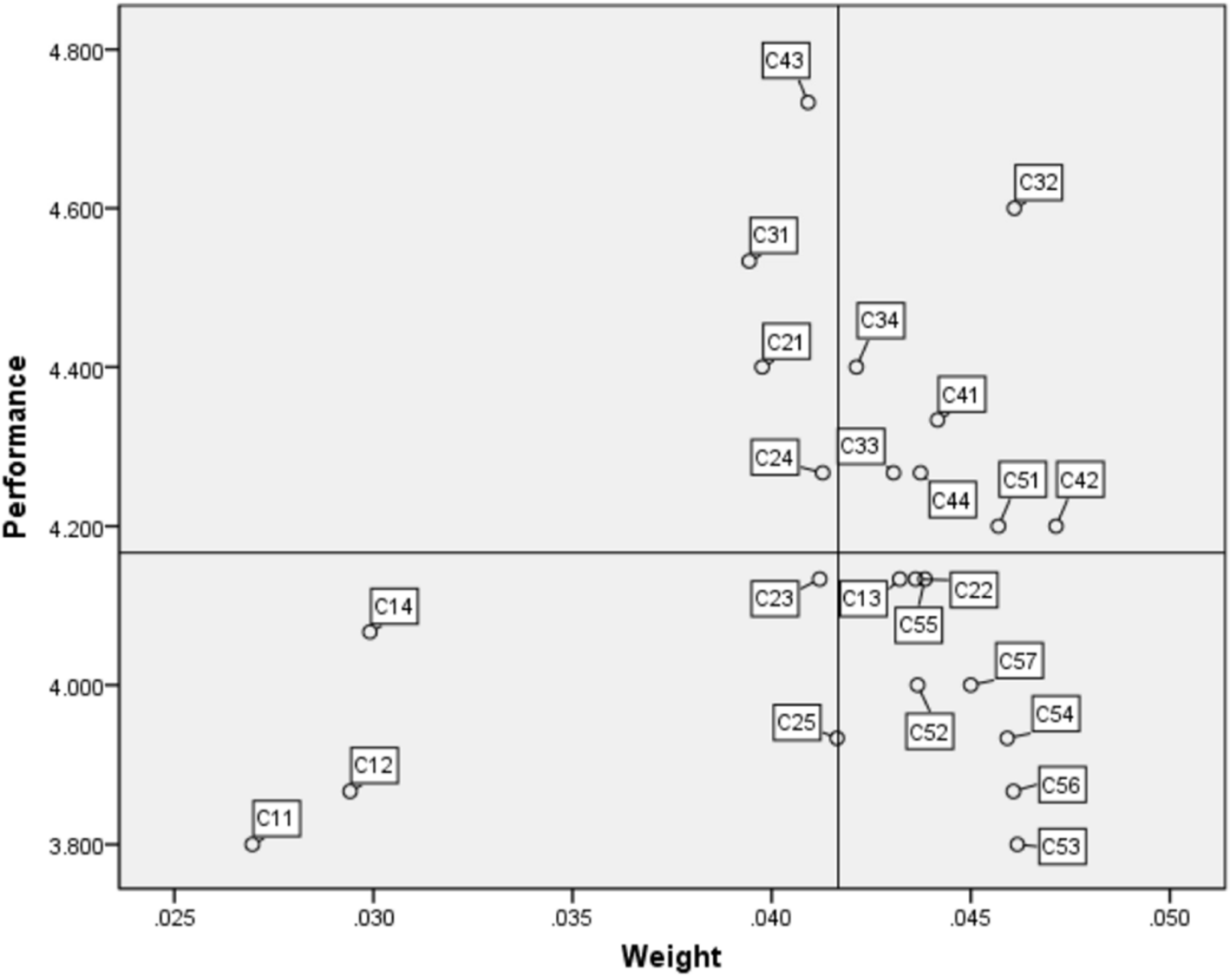
Importance-performance analysis for case hospital

Further, from the perspective of the influence network in Fig. 3, “($C_{5}$)” was the most important influence dimension in the entire training course. Hence, to improve the service quality of the first aid training class for new nurses, the nursing department should focus on transitioning to teaching from the perspective of students, in addition to their professional knowledge and practical experience. Teachers’ professionalism is the foundation; however, effective teaching is based on a demonstration of empathy.

## Limitations

This study had certain limitations. First, it was based on the SERVQUAL theoretical model to design a service evaluation model, thus skewing the perspective. Different research evaluations focus on different viewpoints, and the design of the evaluation index system also differs. Future researchers could study the index system more deeply from different theoretical viewpoints.

Additionally, the results of this study were based on the participants of the case hospital and may not be applicable to other courses or hospitals. In the future, researchers can collect more survey data and explore the operation mechanism behind more realistic phenomena using statistical analysis or machine learning methods. Furthermore, assumptions about experience and empathy are usually difficult to measure accurately by quantitative methods [35, 36], and future researchers can combine qualitative theory with data for in-depth analysis.

## Conclusion

Teachers’ empathy is vital to the learning outcomes of new nurses in emergency nursing training courses. The main reason is that new nurses come from different specialties and lack knowledge and clinical experience in emergency nursing. Therefore, new nurses may encounter many learning obstacles and issues during emergency nursing training. Therefore, teachers should pay special attention to the learning status of new nurses to help them gain knowledge and experience in emergency nursing. Ultimately, each new nurse in the emergency department will be able to complete the training process. When the country faces a major public health emergency in the future, registered nurses and new nurses in the emergency department will be well-equipped to address the needs and problems of local emergency care as quickly as possible.

## Data Availability

The study data and materials are in the custody of the corresponding author and can be made available on reasonable request.
